# Factors Associated with HIV Drug Resistance in Dar es Salaam, Tanzania: Analysis of a Complex Adaptive System

**DOI:** 10.3390/pathogens10121535

**Published:** 2021-11-24

**Authors:** Anneleen Kiekens, Idda H. Mosha, Lara Zlatić, George M. Bwire, Ally Mangara, Bernadette Dierckx de Casterlé, Catherine Decouttere, Nico Vandaele, Raphael Z. Sangeda, Omary Swalehe, Paolo Cottone, Alessio Surian, Japhet Killewo, Anne-Mieke Vandamme

**Affiliations:** 1 Department of Microbiology, Immunology and Transplantation, Rega Institute for Medical Research, Clinical and Epidemiological Virology, Institute for the Future, KU Leuven, 3000 Leuven, Belgium; lara.zlatic0508@gmail.com (L.Z.); georgemsema.bwire@kuleuven.be (G.M.B.); annemie.vandamme@kuleuven.be (A.-M.V.); 2 Department of Behavioural Sciences, Muhimbili University of Health and Allied Sciences, Dar es Salaam 65015, Tanzania; ihmosha@yahoo.co.uk; 3 FISPPA Department, Università degli Studi di Padova, 35139 Padova, Italy; paolo.cottone@unipd.it (P.C.); alessio.surian@gmail.com (A.S.); 4 Department of Pharmaceutical Microbiology, Muhimbili University of Health and Allied Sciences, Dar es Salaam 65013, Tanzania; sangeda@gmail.com; 5 Dar es Salaam Urban Cohort Study, Dar es Salaam 65013, Tanzania; mangaraalli@gmail.com; 6 Department of Public Health and Primary Care, Academic Centre for Nursing and Midwifery, KU Leuven, 3000 Leuven, Belgium; bernadette.dierckxdecasterle@kuleuven.be; 7 Faculty of Economics and Business, Access to Medicine Research Center, KU Leuven, 3000 Leuven, Belgium; catherine.decouttere@kuleuven.be (C.D.); nico.vandaele@kuleuven.be (N.V.); 8 Department of Business Studies, School of Business, Mzumbe University, Dar es Salaam 20266, Tanzania; oswalehe@mzumbe.ac.tz; 9 Department of Epidemiology and Biostatistics, Muhimbili University of Health and Allied Sciences, Dar es Salaam 65001, Tanzania; jkillewo@yahoo.co.uk; 10 Center for Global Health and Tropical Medicine, Unidade de Microbiologia, Instituto de Higiene e Medicina Tropical, Universidade Nova de Lisboa, 1349-008 Lisbon, Portugal

**Keywords:** case study, complex adaptive system, HIV drug resistance, leverage points, systems mapping, Dar es Salaam, Tanzania

## Abstract

HIV drug resistance (HIVDR) is a complex problem with multiple interconnected and context dependent causes. Although the factors influencing HIVDR are known and well-studied, HIVDR remains a threat to the effectiveness of antiretroviral therapy. To understand the complexity of HIVDR, a comprehensive, systems approach is needed. Therefore, a local systems map was developed integrating all reported factors influencing HIVDR in the Dar es Salaam Urban Cohort Study area in Tanzania. The map was designed based on semi-structured interviews and workshops with people living with HIV and local actors who encounter people living with HIV during their daily activities. We visualized the feedback loops driving HIVDR, compared the local map with a systems map for Sub-Saharan Africa, previously constructed from interviews with international HIVDR experts, and suggest potential interventions to prevent HIVDR. We found several interconnected balancing and reinforcing feedback loops related to poverty, stigmatization, status disclosure, self-esteem, knowledge about HIVDR and healthcare system workload, among others, and identified three potential leverage points. Insights from this local systems map were complementary to the insights from the Sub-Saharan systems map showing that both viewpoints are needed to fully understand the system. This study provides a strong baseline for quantitative modelling, and for the identification of context-dependent, complexity-informed leverage points.

## 1. Introduction

Over the past years, Tanzania has made considerable progress towards reaching the global 95-95-95 goals [1]. In August 2020, an estimated 83% of people living with HIV (PLHIV) in Tanzania were aware of their HIV status, of which 90% were on HIV treatment. Of those on treatment, 92% were virally suppressed. In 2019, the HIV prevalence in Tanzania was estimated to be 4.8%. The Tanzanian epidemic consists entirely of the HIV-1 type as no HIV-2 infections have been reported so far [2,3]. In 2019 the WHO reported alarming increases in pre-treatment HIV drug resistance (PDR) with 12 out of 18 reporting countries exceeding the 10% non-nucleoside reverse transcriptase inhibitor (NNRTI) PDR threshold, triggering immediate national action [4]. A recently published study conducted between 2013 and 2019 found a prevalence of 11% PDR among the 801 antiretroviral therapy (ART)-naïve participants from Tanzania, Kenya, Uganda and Nigeria [5]. Among the ART-experienced participants with unsuppressed viral load (VL), resistance rates of 82.5%, 66.7% and 1.8% were reported for NNRTI, nucleoside reverse transcriptase inhibitor (NRTI) and protease inhibitor (PI) mutations, respectively. Another study in Dar es Salaam from 2010, although with a small sample size, found drug resistance mutations in 82.6% of the included therapy-experienced participants [6]. A systematic literature review published by the WHO showed that the prevalence of pre-treatment NNRTI resistance has been increasing the fastest in Eastern Africa, compared to other low- and middle-income regions [7]. These results underline the importance of addressing HIV drug resistance (HIVDR) in order to sustain the progress towards the goal of ending the epidemic by 2030. 

HIVDR is influenced by a multitude of factors which transcend single disciplines and population levels, and which, together, form a complex, multi-layered and interconnected system [8]. Several individual, socio-economic, structural and health care system related factors influencing HIVDR in Tanzania have been described in a literature review by Msongole et al. [9]. Although the diverse factors influencing HIVDR are relatively well studied, preventing HIVDR (including acquired and transmitted drug resistance) in the real world remains difficult [4,10,11,12]. In order to understand and address the underlying challenges of HIVDR there is a need to shift away from the reductionist, linear cause-effect models towards a comprehensive systems approach and study the factors associated with HIVDR as a complex adaptive system (CAS) [13]. A core characteristic of such CAS lies in the understanding that successfully intervening on one element of a system does not guarantee resolving the central problem due to influences of other aspects of the system [14]. Interventions in a complex system ideally require a small shift in one place which has the potential to positively change the whole system. Such places to intervene on are called leverage points and can be divided into shallow and deep leverage points [15,16]. Shallow leverage points, such as parameters and feedbacks, are relatively easy to intervene on, but have a limited effect on the system, whereas interventions at deep leverage points are difficult to accomplish but can result in an extensive change of the system. Interventions at deep leverage points are aimed at changing the underlying structure, goal, or paradigm of the system. Achieving this requires a joint understanding of the system by scientists, stakeholders (including PLHIV) and societal actors, as well as a joint commitment towards supporting the envisioned change. With this study we took a first step in this direction and studied HIVDR in its totality as a CAS of interconnected and interacting factors. Concretely, we aimed to understand how these factors are interconnected with and embedded in the local context of our study area in the Ukonga and Gongolamboto areas of Dar es Salaam, Tanzania [14,17]. We compare this local systems map with one constructed from the knowledge of international experts, developed in a previous study, and discuss the differences and similarities [8]. We also provide a first assessment of potential intervention points. 

## 2. Results

We interviewed 12 PLHIV and 10 local actors in the Dar es Salaam Urban Cohort Study (DUCS) located in the Ukonga and Gongolamboto areas of Dar es Salaam. Of the PLHIV, two were lost to follow-up and ten were engaged in care. Another 10 PLHIV and nine local actors engaged in the validation workshops. The sociodemographic and therapy data of the participants are described in Table 1. Not surprisingly, the majority of participating PLHIV were female, which can be explained by the higher HIV prevalence in women, as well as the lower linkage to care rates in men. Of the 22 PLHIV involved, 18 were on first line dolutegravir-based treatment. The other four were on first-line NVP or EFV-based regimens. Overall, we reached a diverse sample of participants which allowed us to study the factors influencing HIVDR from different angles. Data saturation for the factors influencing HIVDR (elements) was reached after 16 interviews and after about 19 interviews for the connections between those factors (Figures S1 and S2).

**Figure 1:**
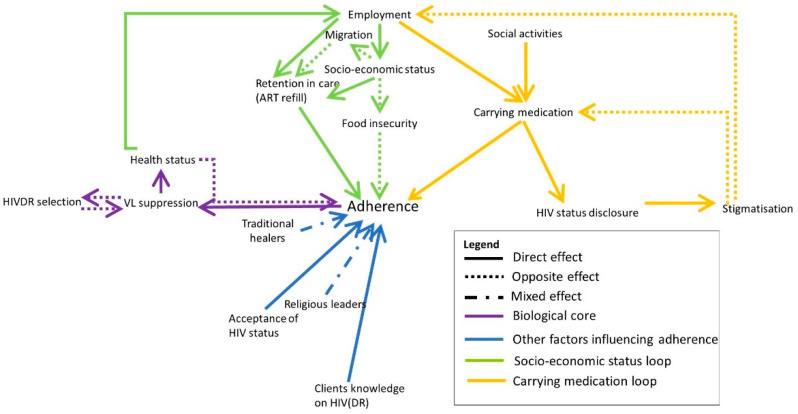
The participants’ perspectives on HIV drug resistance in the study site, reflected in three core loops related to the health status (purple), socio-economic situation (green) and involuntary status disclosure when carrying medication (yellow). Some additional factors influencing HIVDR are indicated in blue. Full arrows indicate that both elements are evolving in the same direction (e.g., A->B: when A increases, B increases as well). Dotted arrows indicate an opposite effect (when A increases, B decreases). Mixed arrows indicate that the effect can be either direct or opposite.

**Figure 2:**
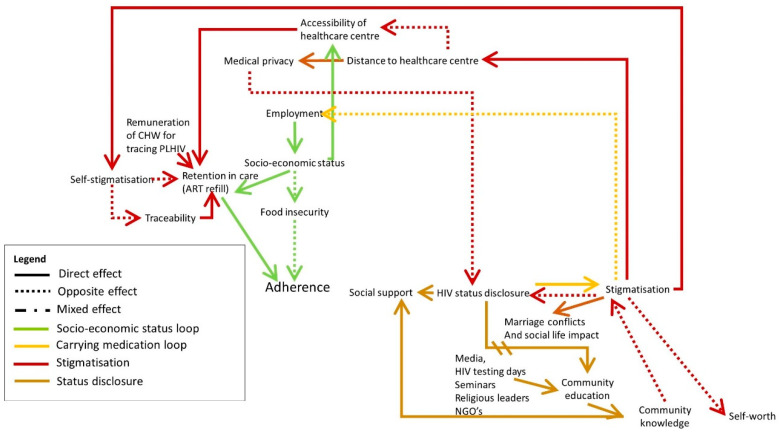
The causes and effects of stigmatisation and HIV status disclosure are indicated in dark red and brown. The arrow with double strikethrough indicates a relationship with a delayed effect. Full arrows indicate that both elements are evolving in the same direction (e.g., A->B: when A increases, B increases as well). Dotted arrows indicate an opposite effect (when A increases, B decreases). Mixed arrows indicate that the effect can be either direct or opposite.

**Figure 3:**
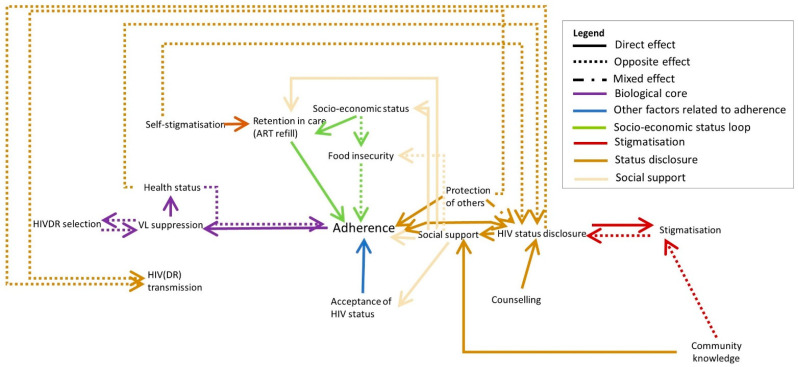
The influences of social support are indicated in beige. Full arrows indicate that both elements are evolving in the same direction (e.g., A->B: when A increases, B increases as well). Dotted arrows indicate an opposite effect (when A increases, B decreases). Mixed arrows indicate that the effect can be either direct or opposite.

**Figure 4:**
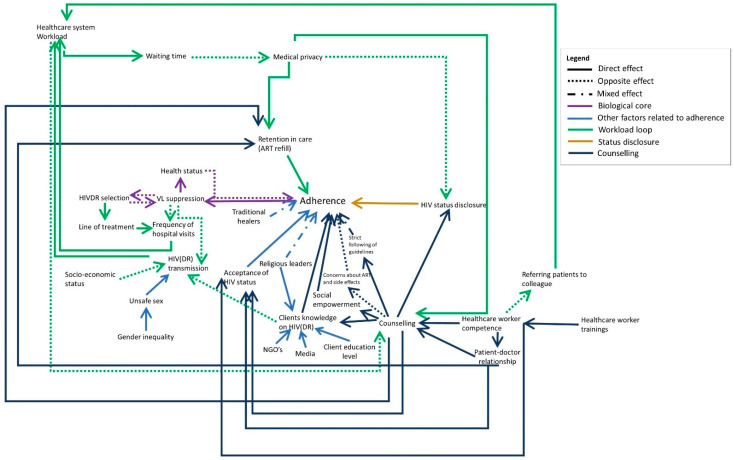
The reinforcing workload loop is indicated in dark green. Full arrows indicate that both elements are evolving in the same direction (e.g., A->B: when A increases, B increases as well). Dotted arrows indicate an opposite effect (when A increases, B decreases). Mixed arrows indicate that the effect can be either direct or opposite.

**Figure 5:**
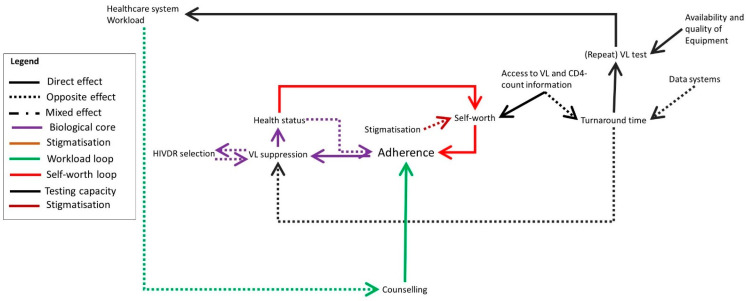
The importance of linking back testing results to the clients, and related practical requirements are indicated in red and black, respectively. Full arrows indicate that both elements are evolving in the same direction (e.g., A->B: when A increases, B increases as well). Dotted arrows indicate an opposite effect (when A increases, B decreases). Mixed arrows indicate that the effect can be either direct or opposite.

**Figure 6:**
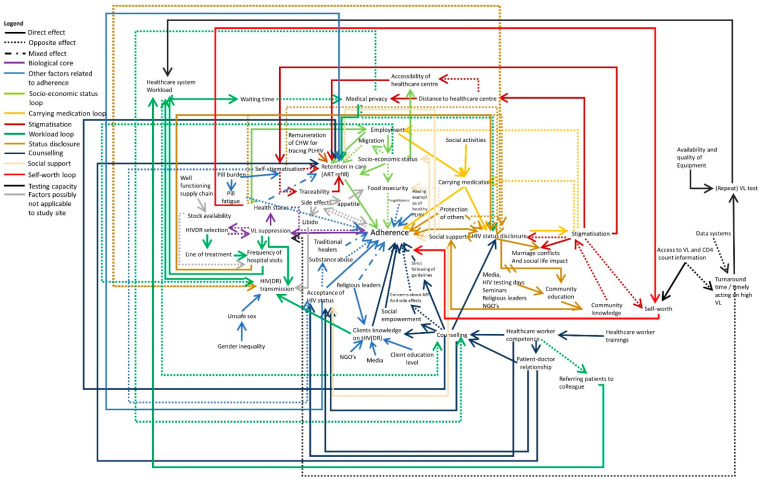
The full system of factors influencing HIVDR in the study site. Additional factors influencing adherence and some elements which are no longer applicable are indicated in blue and grey, respectively. Full arrows indicate that both elements are evolving in the same direction (e.g., A->B: when A increases, B increases as well). Dotted arrows indicate an opposite effect (when A increases, B decreases). Mixed arrows indicate that the effect can be either direct or opposite. See also Figure S3.

Based on the collected data, we developed a systems map representing the factors influencing HIVDR in the Ukonga and Gongolamboto areas in Dar es Salaam as experienced by the local population. The map consists of several interconnected feedback loops which we will describe step by step. In Figure 1, Figure 2, Figure 3, Figure 4 and Figure 5, parts of the system are shown, whereas the complete system is presented in Figure 6. The purple section of Figure 1 represents the biological mechanism of HIVDR selection. HIVDR is selected under selective pressure caused by incomplete VL suppression. A major cause of incomplete VL suppression is suboptimal adherence, here defined as the compliance of PLHIV with their therapy as well as the possibility for them to take their medication daily, thus including both factors that are within and out of their own control. Selection of HIVDR will lead to an increase in opportunistic infections and generally poorer health as a result of an unsuppressed VL. The interviewees described situations in which clients do not believe they are HIV-positive when they do not experience symptoms after testing or who believe they are cured when their health improves and therefore do not see the need to adhere anymore. These clients then re-start taking their ART when they develop symptoms. This may be fuelled by a lack of knowledge about HIV, by the influence of traditional healers or religious leaders who claim to cure HIV, or by the client not accepting their HIV status. A major barrier to adherence in the study site is poverty (Figure 1, green colour). Clients who cannot afford a meal each day, may skip their medication, out of fear of side effects. Clients living in poverty may also have difficulties picking up medication when they do not have money to pay for transportation or when they are offered an employment opportunity on the day of their refill and have to choose between income and medication. One participant described this as follows:

“When I say that money is more important than health, it’s not that health is not important but they depend on each other. It happens that you stayed hungry for three days and failed to take your medication because of the food insecurity. The fourth day someone calls you to go to work and get money, tell me if it were you, what would you do? Would you go to the clinic or to work?”

—PLHIV (Female, 48 years old)

Clients migrating to other parts of Tanzania in search of an income or for other purposes may also experience difficulties remaining in care. The socio-economic aspects of HIVDR are very prominent in the study site as barriers to adherence but also as motivators. The knowledge that when adhering to therapy, one will be in good health, able to work and provide income for the family, drives clients to adhere well, a motivational strategy which is also used by the healthcare workers.

The yellow arrows in Figure 1 illustrate an issue caused by the stigmatized nature of HIV in the community. When joining social activities or travelling for work, some clients do not take their medication with them out of fear of involuntary status disclosure, subsequent stigmatization, and the possibility of losing employment opportunities. 

Participants indicated that stigma and discrimination can have a profound effect on PLHIV’s lives, reflected by the dark red and brown loop in Figure 2. Next to the risk of losing employment, the participants reported that stigma and discrimination can be the cause of marital or familial conflicts, discrimination at social gatherings and general discomfort due to gossip or being treated differently. Moreover, the impact on people’s self-esteem can cause them to self-stigmatise. To avoid that, they often choose not to disclose their status, drop out of care, or become nonadherent. Some even go as far as to give fake contact details to the healthcare staff in order not to be traceable. Others prefer to go to a healthcare centre far from home in order not to be recognised. However, this may come with the challenge of sustainably accessing this healthcare centre for each refill and check-up due to for example financial constraints. The participants indicated that stigma and discrimination can be prevented by educating the community on HIV, its modes of transmission, prevention, treatment and required support. They also expressed the need to encourage the community to appreciate and support PLHIV who disclose their HIV health status. This can be achieved through the media, brochures, and seminars given by NGOs, or for example by religious leaders who have a wide reach. 

Over time, the more PLHIV disclose their status and openly talk about HIV, the more the community will learn about HIV. This increased community education is expected to decrease the stigma surrounding HIV, encouraging more PLHIV to disclose their status and adhere to ART. This is a delayed reinforcing loop.

HIV status disclosure can have positive and negative consequences: on the one hand, stigmatisation can have a profound effect on social life as discussed above. On the other hand, people may receive social support from their family who can help them to adhere and accept their status, or who can help them financially or by providing meals (Figure 3, beige arrows). A person living with HIV may experience both positive and negative consequences and may therefore choose to disclose their status only to a select group of people. Counselling can help to prepare PLHIV to disclose their status. Some participants reported not disclosing their status in order to spare their loved ones from worrying about them. However, the will to protect others may also motivate PLHIV to disclose their status in order to engage in safer sex and to adhere to their medication in order not to infect others. 

Counselling can help PLHIV to accept their HIV status, gain a deeper understanding about HIV and ART and feel socially empowered to ask questions or demand VL tests for instance. In some cases, the health care provider gives very strict guidelines (such as dietary information or the guideline to take the medication strictly at a certain time) which may discourage the client to take the ART when they cannot meet these requirements.

“… However, we shouldn’t miss the nutrients they recommended in our foods. … I don’t know things like finger millet and others, we are missing them in our foods because we can’t afford to get them, we are missing the nutrients. … For instance, the ones with [financial] ability. Vegetables, small fried fishes aren’t bad. They told us not to use beef, it isn’t good that’s what they said. For instance, they told me an old man like me what I should eat is like pig’s meat, chicken and fishes. Now things I am able to get in most cases are green vegetables and stiff porridge. You see how it is hard. … They told me so, but they didn’t tell me the reasons. They told me that I shouldn’t prefer using beef.”

—PLHIV (Male, 34 years old)

Elements important for good counselling sessions that arose from the interviews include: medical privacy (in some cases, there are multiple clients in the doctor’s office or the door is left open), well-trained healthcare workers and community health workers (CHW) who are able to answer the clients’ questions and who have a caring attitude, and a good client-provider relationship (Figure 4, dark blue). Participants also indicated that this could help clients to accept their HIV status. 

Another important factor is the workload of the healthcare centre. Both PLHIV and local actors indicated that at times the healthcare centre is overburdened, and healthcare providers do not have enough time to provide thorough counselling for all clients, which may impact its efficiency. 

The healthcare system workload increases when PLHIV have to visit the hospital more frequently because they have an unsuppressed VL or developed drug resistance, or when HIV(DR) is transmitted in the community and more people have to enrol in care. When healthcare staff are not sufficiently trained to handle certain cases or answer all questions of the client, they may have to refer the client to other colleagues, therefore also increasing their workload. 

Next to decreased counselling efficiency, a high healthcare system workload also increases the waiting time at the healthcare centre which may lead to PLHIV not picking up their medication as they are afraid of being recognised by other people at the healthcare centre. 

The healthcare system workload loop (Figure 4, dark green) is a reinforcing loop in which the consequences of high workload (decreased counselling quality and therefore a decreased adherence) will eventually lead to an even higher workload.

The following reinforcing loop is indicated in red in Figure 5. Having access to information about one’s health status, such as VL and CD4 count information, especially when the client is doing well, contributes to the client’s feeling of self-esteem. Clients are proud of their good health and are congratulated by healthcare staff, which motivates them to continue adhering. In black, we indicated the impact of the VL testing organization on the system. Test results sometimes arrive with a delay, or not at all because of which the test has to be repeated, further increasing workload. Possible causes of this are a lack of equipment for testing and a lack of uniform electronic data systems to facilitate sharing the results.

Figure 6 shows the full system of all identified factors influencing HIVDR in the study site. Additional to what is described above, other factors influencing adherence are substance abuse (possibly stemming from poor acceptance of one’s HIV status), forgetfulness or pill fatigue as illustrated by the interview quote below. The burden of having to take medication each day for the rest of one’s life may contribute to self-stigmatisation and may on its own be a reason to skip the medication from time to time. Although the first line ART in the study site consists of one pill per day, usually more medication needs to be taken such as medication for opportunistic infections.

“Truly, you can swallow the drugs and there are times you get tired of taking them and say let me skip them today. You can stop for a day; you just say today I am resting. … Only one day, I am scared to skip them for two days because that’s when you are told viruses increase in one day if you skip. … Honestly, for instance for the drugs which I was given for three months. I can rest for one day. … Ahh per three months I only rest once”.

—PLHIV (Female, 39 years old)

In light grey, two elements are added which are no longer applicable for the adult population in our study site. The participants reported relatively little supply issues in the study area and if needed the healthcare centres reorganize themselves and give half supplies to the clients so that everyone can be served until they have restocked. Additionally, the side effects are of lesser concern since first line treatment has been switched from tenofovir/lamivudine/efavirenz (TLE) to tenofovir/lamivudine/dolutegravir (TLD). It is important to note that side effects can demotivate clients from adhering to therapy directly, but clients can also experience being hungry after taking the medication and therefore skip the medication when they know they will not be able to satisfy their increased appetite. Some clients also report an increased libido after taking the medication and indicated that this increases the transmission risk.

**Figure 7:**
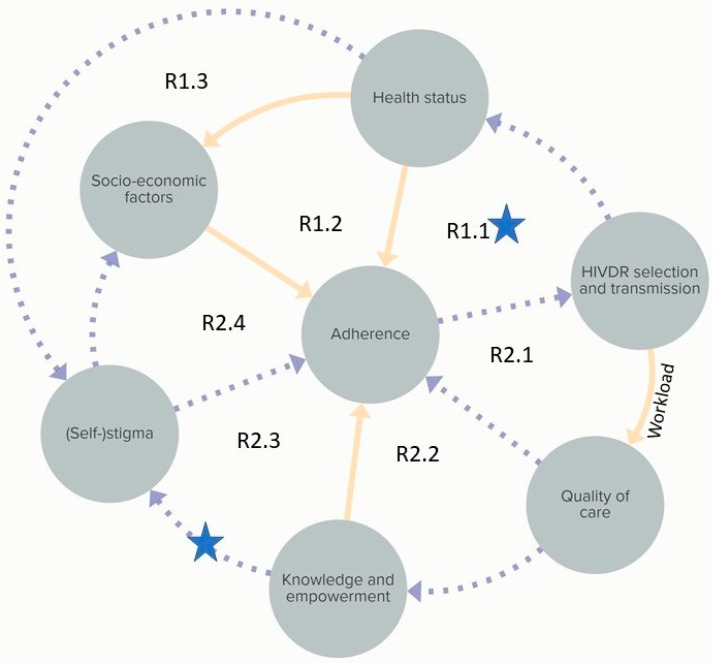
Summary figure of the CAS of factors associated with HIVDR in the study area. Seven reinforcing loops and two leverage points (blue stars) are indicated. The third leverage point is at the structural level and is therefore not visualised here. Individual reinforcing loops are indicated as R1.1 to R2.4. Full arrows indicate that both elements are evolving in the same direction (e.g., A->B: when A increases, B increases as well). Dotted arrows indicate an opposite effect (when A increases, B decreases).

While the above systems map represents the CAS in detail, Figure 7 summarizes the system into seven core loops representing the main mechanisms behind HIVDR in the study site. In the following paragraph the core loops and three identified leverage points are discussed. R1.1 is a reinforcing loop through which PLHIV are motivated to keep adhering to the ART because of their improved health status. The first, shallow level leverage point identified is the strengthening of this loop, for example through motivation by healthcare workers. Reinforcement of R1.1 will automatically weaken R2.1 and R2.2 which represent the effects of an increased healthcare system workload when adherence levels are not sufficient. The decreased time for counselling and other support for PLHIV will lead to a further decrease in adherence levels. Furthermore, R1.1 reinforcement would strengthen R1.2, which results in improved adherence through increased socio-economic opportunities. It would also decrease R1.3 as healthy looking PLHIV tend to be less stigmatized by others and by themselves. The second, also shallow leverage point is to weaken R2.3 and R2.4 which represent a decreased adherence through stigmatization and decreased socio-economic opportunities, respectively. This could be done by providing community education, potentially through religious leaders, community leaders or traditional healers, who have a wide range. 

The third leverage point is identified at the design level and is therefore considered a deep leverage point. Based on the combined needs for economic support, education on HIV(DR) and improving the mental well-being of PLHIV, we propose the organization of microfinance groups specifically for PLHIV. Microfinance groups are informal financial support groups where members are educated on entrepreneurship, contribute a monthly amount of money and have the opportunity to request a loan from the group. These groups may be a platform for PLHIV to combine their economic support group with peer support-like activities such as education sessions on HIV(DR) and practical and psychological support [18]. Although the economics of microfinance groups for PLHIV have been described in the literature, more research remains to be conducted on the effect on health outcomes [19,20].

The summary system in Figure 7 is influenced by several other factors which are here considered external and therefore not represented. These are, for example, supply chain related factors, testing capacity and ART properties. 

## 3. Discussion

In this study, we gained insight into the complexity of HIVDR in the Ukonga and Gongolamboto areas of Dar es Salaam by developing a model representing the CAS of its interconnected factors, together with local actors and PLHIV. It is important to note that our aim was to understand the CAS of factors influencing HIVDR through the mental models of the people most affected by it. Therefore, the model does not represent one fixed reality but rather an interconnected network of elements influencing HIVDR, which are constantly evolving over time, and which are highly dependent on context.

Three leverage points were identified based on the insights provided by our systems map. The first, shallow, leverage point aims at reinforcing the motivation to adhere to therapy, for instance through the encouragement of positive health outcomes. The second also shallow one, aims at decreasing stigmatization by strengthening community education. The third identified leverage point is at a deeper level and requires the restructuring of certain aspects of care through combining microfinance and peer support groups for PLHIV. Our work provides valuable insights at the systems level which, after strengthening of the healthcare system viewpoint, can be used to design and test interventions at these leverage points.

In addition to the identified leverage points, we obtained some other system-level insights. First, our data clearly showed the impact of psychological wellbeing on the dynamics of the HIVDR system as also described extensively by Zlatić et al. [21]. In particular, stigmatization was found to be the driver of several important feedback loops. Second, at the healthcare system level, we found that some counsellors give very strict guidelines to their clients which are ill-adapted to their life circumstances. These are failing to convey their purpose, and therefore sometimes work counterproductively. Clients may refrain from taking their medication if they do not find the advised type of food or if they come home one hour late. Future seminars on HIVDR for healthcare workers may need to be revised to refocus on the objective of the counselling sessions (preventing HIVDR and ensuring good health of PLHIV) rather than on the individual rules they have to follow. Third, at the community level, we found a delayed reinforcing feedback loop, indicating that PLHIV openly disclosing and discussing their HIV status are conducting a type of community education. This can reduce community stigmatization over time, encouraging more PLHIV to disclose their status. Previous studies have shown the correlation between knowledge and HIV related stigma [22,23]. One study in South Africa found that a decrease in stigma was associated with an increase in knowledge over a period of four years [22]. To identify the tipping point at which this reinforcing loop is kicked into action additional research is needed. 

To explore the contents of our systems map beyond the local level, we compared it with a systems map of factors influencing HIVDR for Sub-Saharan Africa, which was informed by experts and developed using the same methodology [8]. Overall, the content of the systems maps remains largely similar. As can be expected, however, the expert systems map contained more extensive information at the healthcare system level and the local map goes into more detail at the personal level. A notable difference is that, whereas in the expert map the economic factor food insecurity was considered to be important but external to the system, it became clear that at the local level those factors were at the very core of the system, forming daily barriers to adherence for PLHIV. This shows that in order to fully understand the CAS of HIVDR, the viewpoints of PLHIV, actors and experts, as well as those groups at the local and broader geographical level need to be integrated.

A shortcoming of this study is its timeframe as two important events happened: (1) at the time of data collection the healthcare centres in the study site had just switched their ART regimens from TLE to TLD, a therapy which evokes less side-effects and which has a lower chance of provoking mutations in the virus and (2) between the data collection and validation the world was hit by the COVID-19 pandemic which, for a period of time brought a number of changes to the system. From March until July 2020, all PLHIV in the study area were given ART for six months instead of the usual one or three months, wearing face masks was obligatory in the healthcare centre, which caused problems for clients who could not afford them and transportation fees increased due to strict rules for seat capacity of commuter buses. While further research is needed to clarify the impact of these interruptions on the HIVDR prevalence in the population, our systems map can help to understand how these measures may have impacted the adherence level of PLHIV. Moreover, the systems mapping method described can be used to study the impact of the COVID-19 pandemic on other aspects on the healthcare system, to study other public health problems, or to be transferred to study HIVDR in other study sites.

## 4. Materials and Methods

### 4.1. Study Design

An iterative systems mapping design was used to visualize and analyse the CAS of factors associated with HIVDR in our case study site in Dar es Salaam, Tanzania. Qualitative methods were used for data collection and analysis. The systems analysis and identification of leverage points were based on a systems thinking inspired analysis guide [24]. 

### 4.2. Study Site and Participants

The study was conducted at the DUCS site in the Ukonga and Gongolamboto administrative wards, Illala district, Dar es Salaam region, Tanzania. The DUCS follows more than 100,000 residents from more than 20,000 households and collects sociodemographic and other data on a six-monthly basis [17]. This study site was chosen because of the rich data available which may support future intervention designs. We included three types of stakeholders in this study, each representing a different perspective: local experts, local actors, and PLHIV. Local experts were people with professional expertise on HIVDR, based in Tanzania. For the purpose of this study, local actors are defined as people who have good insights in the daily lives of the local citizens and who, through their job, status or daily activities are able to make a positive impact in their society. The local actors were selected with the aim of including a range of people who could provide us with insights about HIV in the community from diverse angles in order to create an overview that is as comprehensive as possible. PLHIV in several stages of their treatment, on different therapy regimens and with varying treatment-adherence levels were selected purposefully and recruited by research assistants of the DUCS. 

### 4.3. Data Collection Procedures

The systems map was developed in three phases (Table 2). During the preparation phase we organized a workshop with local experts to discuss factors influencing HIVDR in our study site. During this meeting we started from a Sub-Saharan systems map based on knowledge from international experts, developed in previous research and adapted this map to the local situation. This adapted map served as a basis to design the semi-structured interview guides and was not used further in data analysis. This way, the CAS of HIVDR in our study site was constructed anew from the interview data, truly allowing the perspectives and mental models of the local inhabitants to form the map, without the influence of previous research.

### 4.4. Semi-Structured Interviews

The first draft of the systems map was designed based on semi-structured interviews with PLHIV and local actors at DUCS in the Ukonga and Gongolamboto areas in Dar es Salaam, Tanzania. Semi-structured interviews do not consist of a set of rigorous questions but rather use a set of common themes to be explored with all the participants. This type of interview allows new themes to come up and be explored, based on the interviewee’s answers.

The participants were called on their cell-phone and invited for a face-to-face interview at the DUCS office in the local community centre located in the Ukonga area. This location is neutral and not linked to any activities involving PLHIV and was therefore chosen to avoid stigmatization of the participants. The interviews were held in Kiswahili by I.M., a local social scientist and participants were reimbursed for their transportation costs. Each interview session lasted for about forty-five minutes. The interviews were audio recorded after seeking consent from study participants, transcribed verbatim and translated into English.

The semi-structured interview guide was informed by the expert meeting and designed by A.K., A.V. and I.M. with the aim of capturing the deeper factors influencing HIVDR in the DUCS area. After each interview day I.M., A.K. and A.V. met to debrief the interviews and the interview guide was adapted according to the insights gained. After a first analysis of the interviews, a selection bias was noted as only participants enrolled in care were interviewed. In order to have a more diverse perspective on the factors influencing HIVDR in the study area, two additional participants who had not been attending healthcare services regularly in the past months were recruited and interviewed during a phone conversation. Interviews were conducted until data saturation was reached. For the purpose of this study, data saturation was defined as the moment in which no new elements or connections are discovered in two consecutive interviews (Table S1).

### 4.5. Data Analysis

The analysis of the semi-structured interviews was conducted by two researchers (L.Z.) and (A.K.) with a combined background in psychology, biomedical science and systems thinking. The method used was inspired by the QUAGOL method [25]. After each interview, a technical report was written, containing all the specifics needed for a full comprehension of the data in their specific context. In order to ascertain a correct interpretation and cultural understanding of the transcripts, they were each individually discussed in a series of meetings between A.K., L.Z. and I.M.

For each transcript, a respective systems map was made, visualizing the factors influencing HIVDR mentioned in the interview and the connections between those factors. Seven interviews with PLHIV and five local actor interviews were schematized and coded by A.K. The other five interviews with PLHIV and five local actor interviews were schematized by both A.K. and L.Z. and the interviews were coded by L.Z. Possible differences were discussed until a consensus was found. In a next phase the separate schemes were merged together into one comprehensive systems map containing all the codes extracted from the interviews. The systems map was designed with the online mapping tool KUMU, which facilitates the visualization and analysis of the map, as different types of data can be stored behind the elements and connections [26]. Though here described linearly, the coding and mapping was an iterative process in which the interviews were re-read at several points in time, codes were revised throughout discussions between the researchers and findings were constantly compared with insights from previously analysed interviews.

### 4.6. Validation

A validation round of the systems map was held in two workshops, one with PLHIV and one with local actors, organized in February and March 2021. The discussion was organized around six central areas of the systems map. The participants discussed the model, changes in the model since the first data collection, and possible interventions. The workshops were organized in the form of a focus group discussion, conducted in Kiswahili. The workshops were recorded, transcribed, and translated into English and they were coded and analysed following the same method as for the semi-structured interviews above.

## 5. Conclusions

We successfully modelled the CAS of factors influencing HIVDR in the Ukonga and Gongolamboto areas of Dar es Salaam, Tanzania. The model provides a detailed understanding of the mechanisms that locally drive HIVDR, based on which we suggested three local leverage points. Together this forms a strong basis for the design of sustainable, complexity-informed interventions, tailored to the local context of the study site.

## Figures and Tables

**Table 1:** Sociodemographic and therapy data of the participants of the interviews and validation workshops.

	PLHIV (N = 22)	Local Actors (N = 19)
Average age (year)	40 (21–56)	49 (33–73)
Gender		
Male	18% (4)	63% (12)
Female	82% (18)	37% (7)
Education		
No degree	14% (3)	16% (3)
Primary education	77% (17)	26% (5)
Secondary education	9% (2)	16% (3)
Higher education	0% (0)	42% (8)
Occupation		
Employed	64% (14)	100% (19)
Unemployed	36% (8)	0% (0)
Years of experience in local actor roll		
<5	/	16% (3)
5–10	/	21% (4)
≥10	/	58% (11)
Time since first positive HIV test		
≤1 year	14% (3)	/
2–5 years	36% (8)	/
>5 years	50% (11)	/
Time since start of treatment		
≤1 year	18% (4)	/
2–5 years	36% (8)	/
>5 years	45% (10)	/

**Table 2:** Overview of the different activities and participants in the project.

Phase	Activity	Participants	Purpose
Preparation	Expert meeting + field visit (June 2019)	10 Tanzanian HIVDR experts	Discussion on factors influencing HIVDR in the Tanzanian context, informed by a systems map previously developed with Sub-Saharan African HIVDR experts. This meeting informed our semi-structured interview guide.
Data collection	Semi-Structured interviews (June 2019–February 2020)	12 PLHIV and 10 Local actors	Forming a detailed understanding of the perspectives of PLHIV and local actors on the CAS of factors influencing HIVDR in the study site.
Validation	Workshops (February–March 2021)	10 PLHIV and 9 Local actors	Validating the systems map developed based on the semi-structured interviews and brainstorming about possible interventions for preventing HIVDR.

## Data Availability

All data generated or analyzed during this study is included in this published article and its supplementary information files.

## Supplementary Materials

The following are available online at https://www.mdpi.com/article/10.3390/pathogens10121535/s1, Figure S1: data saturation elements, Figure S2: data saturation connections, Figure S3: Systems map representing the CAS of factors related to HIVDR in the study site, Table S1: table containing all elements and connections.
