# Supplementary material for: Factors Associated with HIV Drug Resistance in Dar es Salaam, Tanzania: Analysis of a Complex Adaptive System
Source: Pathogens. 2021 Nov 24;10(12):1535. doi: 10.3390/pathogens10121535 (PMC8707982; doi:10.3390/pathogens10121535)
Supplement: Supplementary file 1 [file pathogens-10-01535-s001.zip › Figure S1 and S2.pdf]

Supplementary Materials

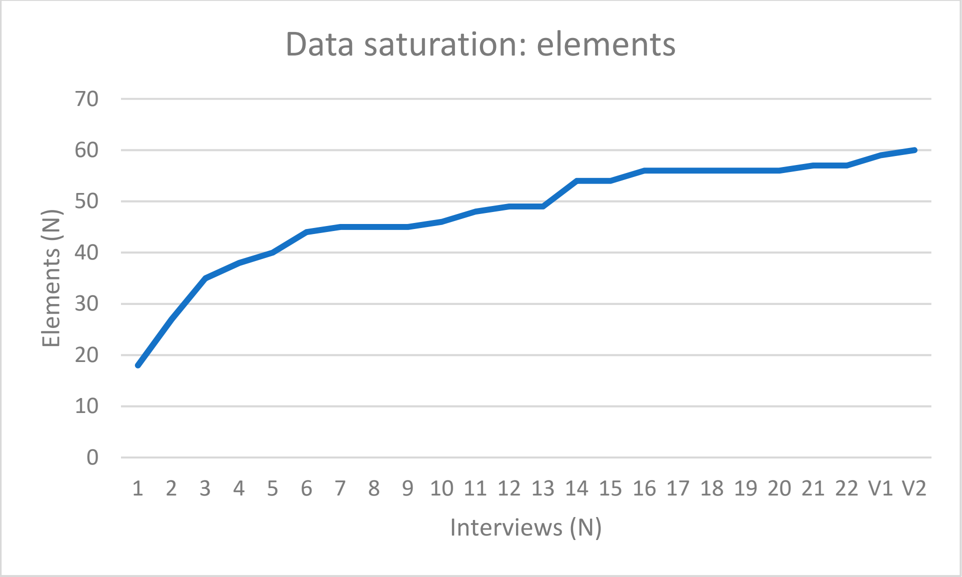

*Figure S1: **data saturation elements**. Data saturation curve of the number of elements after each interview and workshop. V1 and V2 stand for the validation workshops.*

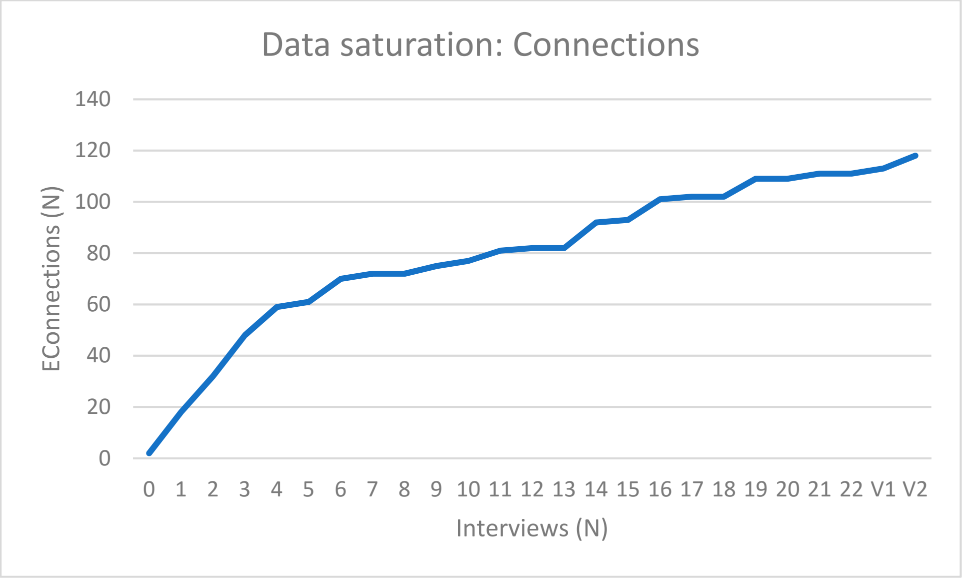

*Figure S2: **data saturation connections.** Data saturation curve of the number of connections after each interview and workshop. V1 and V2 stand for the validation workshops.*
