# Supplementary material for: Factors Associated with HIV Drug Resistance in Dar es Salaam, Tanzania: Analysis of a Complex Adaptive System
Source: Pathogens. 2021 Nov 24;10(12):1535. doi: 10.3390/pathogens10121535 (PMC8707982; doi:10.3390/pathogens10121535)
Supplement: Supplementary file 1 [file pathogens-10-01535-s001.zip › Figure S3.pdf]

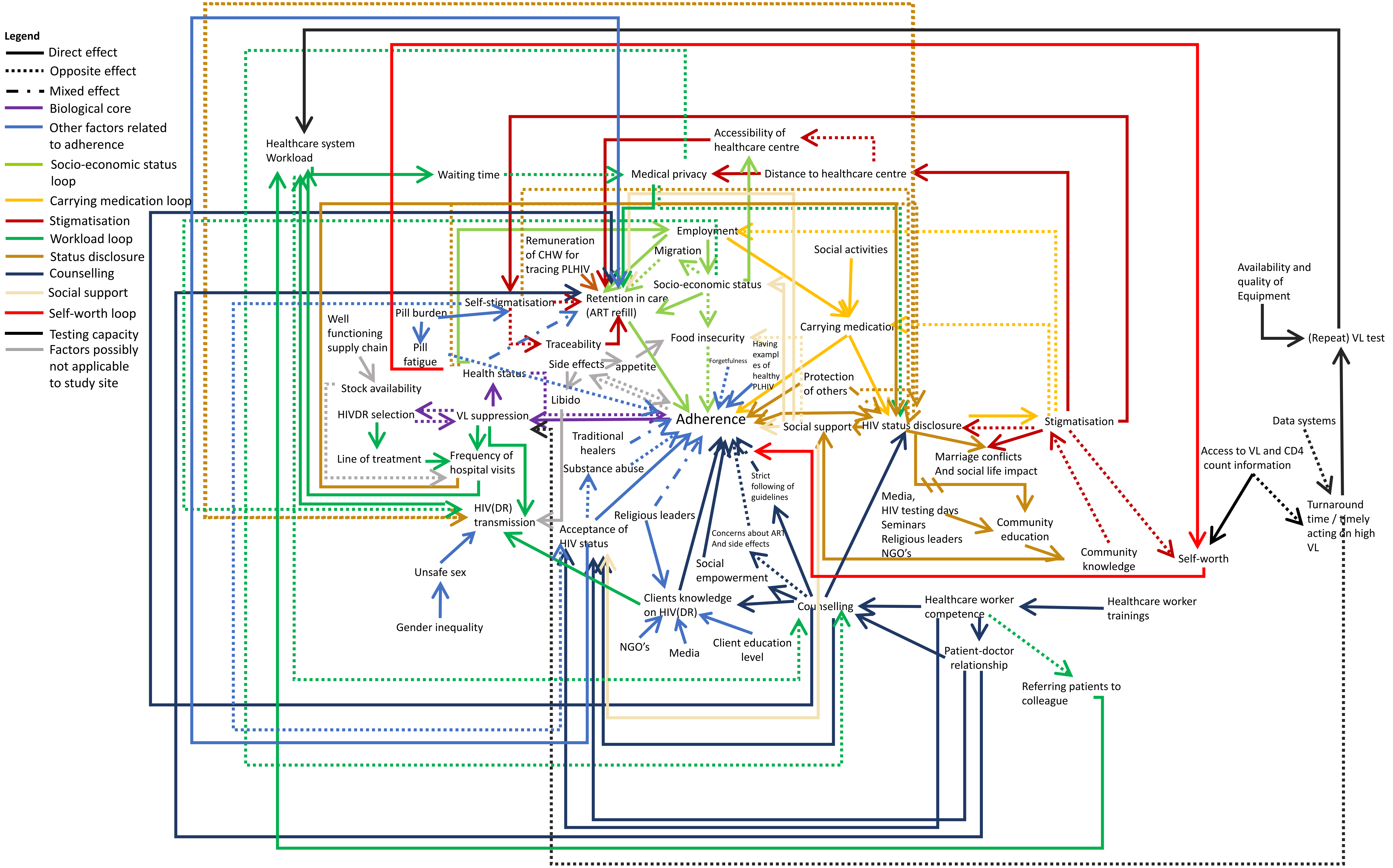

Figure S3: Systems map representing the CAS of factors related to HIVDR in the study site.
